# Use of the CHA2DS2-VASc Score for Risk Stratification of Hospital Admissions Among Patients With Cardiovascular Diseases Receiving a Fourth-Generation Synchronous Telehealth Program: Retrospective Cohort Study

**DOI:** 10.2196/12790

**Published:** 2019-01-31

**Authors:** Jen-Kuang Lee, Chi-Sheng Hung, Ching-Chang Huang, Ying-Hsien Chen, Pao-Yu Chuang, Jiun-Yu Yu, Yi-Lwun Ho

**Affiliations:** 1 Telehealth Center National Taiwan University Hospital Taipei Taiwan; 2 Division of Cardiology Department of Internal Medicine National Taiwan University College of Medicine and Hospital Taipei Taiwan; 3 Department of Laboratory Medicine National Taiwan University College of Medicine and Hospital Taipei Taiwan; 4 Department of Nursing National Taiwan University Hospital Taipei Taiwan; 5 Department of Business Administration College of Management National Taiwan University Taipei Taiwan

**Keywords:** CHA2DS2-VASc score, fourth-generation synchronous telehealth program, hospitalization, cardiovascular disease

## Abstract

**Background:**

Telehealth programs are generally diverse in approaching patients, from traditional telephone calling and texting message and to the latest fourth-generation synchronous program. The predefined outcomes are also different, including hypertension control, lipid lowering, cardiovascular outcomes, and mortality. In previous studies, the telehealth program showed both positive and negative results, providing mixed and confusing clinical outcomes. A comprehensive and integrated approach is needed to determine which patients benefit from the program in order to improve clinical outcomes.

**Objective:**

The CHA_2_DS_2_-VASc (congestive heart failure, hypertension, age >75 years [doubled], type 2 diabetes mellitus, previous stroke, transient ischemic attack or thromboembolism [doubled], vascular disease, age of 65-75 years, and sex) score has been widely used for the prediction of stroke in patients with atrial fibrillation. This study investigated the CHA_2_DS_2_-VASc score to stratify patients with cardiovascular diseases receiving a fourth-generation synchronous telehealth program.

**Methods:**

This was a retrospective cohort study. We recruited patients with cardiovascular disease who received the fourth-generation synchronous telehealth program at the National Taiwan University Hospital between October 2012 and June 2015. We enrolled 431 patients who had joined a telehealth program and compared them to 1549 control patients. Risk of cardiovascular hospitalization was estimated with Kaplan-Meier curves. The CHA_2_DS_2_-VASc score was used as the composite parameter to stratify the severity of patients’ conditions. The association between baseline characteristics and clinical outcomes was assessed via the Cox proportional hazard model.

**Results:**

The mean follow-up duration was 886.1 (SD 531.0) days in patients receiving the fourth-generation synchronous telehealth program and 707.1 (SD 431.4) days in the control group (*P*<.001). The telehealth group had more comorbidities at baseline than the control group. Higher CHA_2_DS_2_-VASc scores (≥4) were associated with a lower estimated rate of remaining free from cardiovascular hospitalization (46.5% vs 54.8%, log-rank *P*=.003). Patients with CHA_2_DS_2_-VASc scores ≥4 receiving the telehealth program were less likely to be admitted for cardiovascular disease than patients not receiving the program. (61.5% vs 41.8%, log-rank *P*=.01). The telehealth program remained a significant prognostic factor after multivariable Cox analysis in patients with CHA_2_DS_2_-VASc scores ≥4 (hazard ratio=0.36 [CI 0.22-0.62], *P*<.001)

**Conclusions:**

A higher CHA_2_DS_2_-VASc score was associated with a higher risk of cardiovascular admissions. Patients accepting the fourth-generation telehealth program with CHA_2_DS_2_-VASc scores ≥4 benefit most by remaining free from cardiovascular hospitalization.

## Introduction

Cardiovascular diseases remain the biggest health burden worldwide [1]. Telemedicine can be used to monitor disease and treat patients in real-time. Previous studies showed that patients with cardiovascular disease accepting telehealth medicine had better control of vascular risk factors such as hypertension, diabetes mellitus, and dyslipidemia [2]. Both Spyros et al and Sally et al reported that telemedicine was an important prognostic factor for reducing all-cause mortality in patients with congestive heart failure [3,4]. We have shown that the fourth-generation telehealth program—an internet-based, synchronized, disease-management program providing an immediate response—could lower mortality as compared to the control group [5]. However, Takahashi et al found that telemonitoring did not lead to fewer hospitalizations or emergency department visits [6]. A review article suggested that telemedicine should be carefully evaluated and applied to patients who benefit with improved clinical outcomes [7].

The European Society of Cardiology has published atrial fibrillation-management guidelines in 2016 to advocate using the CHA_2_DS_2_-VASc (congestive heart failure, hypertension, age >75 years [doubled], type 2 diabetes mellitus, previous stroke, transient ischemic attack or thromboembolism [doubled], vascular disease, age of 65-75 years, and sex) score as a predictive scoring model for stroke in patients with atrial fibrillation [8]. In addition, the American Heart Association has already released similar recommendations for treatment of patients with atrial fibrillation in 2014 [9]. Based on the guidelines, patients with one or more stroke risk factors (ie, a CHA_2_DS_2_-VASc score ≥1 in men or ≥2 in women) are at a higher risk for future stroke events, which is a dosage effect. Oral anticoagulation is recommended or preferred for these patients with atrial fibrillation. Furthermore, Mitchell et al showed that CHA_2_DS_2_-VASc scores could predict the incidence of stroke or transient ischemic attack in a population of over 20,000 patients with acute coronary syndrome without atrial fibrillation [10]. This scoring system can also predict various categories of cardiovascular hospitalization events other than cerebrovascular accident events; the scores themselves were a composite of inflammatory risk factors [11]. However, no studies have thus far addressed the utility of CHA_2_DS_2_-VASc scores to stratify patients via a telehealth program.

Here, we aimed to investigate the relation between CHA_2_DS_2_-VASc scores and cardiovascular admission in patients receiving a fourth-generation telehealth program. In addition, we stratified patients by CHA_2_DS_2_-VASc score and examined the effect of the telehealth program on these scores and clinical outcomes.

## Methods

### Study Design

This was a single-center, clinical retrospective epidemiologic study that was approved by the Institutional Review Board of National Taiwan University Hospital, Taipei, Taiwan. All clinical managements of patients in the telehealth program were performed in accordance with relevant guidelines and regulations.

### Patient Selection

The study was conducted from October 2012 to June 2015 at the Telehealth Center of the hospital by the Taiwan ELEctroHEALTH Study Group (TELEHEALTH Study Group). Patients older than 20 years diagnosed with chronic cardiovascular diseases and receiving the telehealth program at our telehealth center were enrolled as the study group. The telehealth-care program is a self-pay service in our hospital, which is not reimbursed by health insurance. Because the patients needed to pay for the service and receive long-term follow-up, we only included patients who were above 20 years old in this study. The decision of receiving the telehealth program depended on the patients or their caregivers. Chronic cardiovascular diseases included coronary artery disease, myocardial infarction, heart failure, peripheral artery disease, stroke, and hypertension. The control group included participants who visited our cardiovascular center during the same period but did not participate in the telehealth care program (received usual care only). The exclusion criteria in this study (for both telehealth group and control group) were age <20 years, absence of any one of the abovementioned chronic cardiovascular diseases, and no follow-up in our hospital.

### Telehealth Care Program

The fourth-generation telehealth program at our center is a synchronized and integrated remote management program for chronic diseases. The internet-based platform was developed by the Graduate Institute of Biomedical Electronics and Bioinformatics, National Taiwan University, Taiwan. The details of this program have been reported previously [12]. Briefly, this telehealth program provides the following services: biometric data including single-lead electrocardiography, blood pressure, heart rate, and oximetry are transferred from patients to our telehealth center daily and on demand; nurse case managers telephone patients daily on demand for communication and health promotion; full-time nurse case managers and cardiologists are in charge of care 24 hours per day; and long-term medication and management are discussed with the patients’ primary care physician after acute events. This telehealth program bridges acute and home care and emphasizes on education, prevention, and early detection of clinical deterioration. The clinical information including CHA_2_DS_2_-VASc scores were relayed to the cardiology specialist who made the final judgment and suggestions regarding care.

### Usual Care

Patients in the control group received the usual care provided by the primary care physicians at our cardiovascular center according to updated guidelines including, but not limited to, the American Heart Association’s guidelines for lifestyle modification and primary prevention to reduce cardiovascular risk, guidelines for the management of stable ischemic heart disease, and the American Diabetes Association’s guidelines for the management of diabetes. Patients made routine outpatient department visits (once every 3 months) to their primary care physicians. There was no contact between the telehealth center and patients receiving usual care.

### Data Collection

All demographic and clinical data were obtained from the electronic database of the hospital. The CHA_2_DS_2_-VASc score was calculated retrospectively according to documentation of the electrical medical chart for congestive heart failure, hypertension, age >75 years (doubled), type 2 diabetes mellitus, previous stroke, transient ischemic attack or thromboembolism (doubled), vascular disease, age of 65-75 years, and sex category. The calculation is not yet an automated process in our Web-based telehealth program. The diagnosis of a chronic disease was based on the electronic database. The discharge diagnosis was used if there was disagreement between outpatient and discharge diagnoses. The follow-up data were acquired from the electronic database of our hospital. The primary outcome of this study was hospitalization for cardiovascular events including acute coronary syndrome, peripheral artery disease, stroke, transient ischemic attack, congestive heart failure, atrial fibrillation, and sudden cardiac death. The end date of follow-up was the September 30, 2016.

### Statistical Analysis

Normally distributed data were displayed as the mean (SD), and data were compared within the study group using the *t* test of variance. Nonnormally distributed continuous data were displayed as the median (interquartile range) and were compared in the study group using the Kruskal-Wallis analysis. The distribution of categorical variables was compared in the study group using the chi-squared test. There were no missing values in the basic variable collections in our cohort.

Kaplan-Meier curves were used to estimate survival rates of hospitalization for cardiovascular events, and a log-rank test was used to compare risks among the study groups. We further stratified the subjects according to the CHA_2_DS_2_-VASc scores of 0-3 and 4-8. To evaluate the independent effect of the telehealth program on the risks of hospitalization for cardiovascular events, we used a multivariable Cox proportional hazard model with adjustment for prespecified clinical characteristics including age >80 years, female gender, diabetes mellitus, hypertension, dyslipidemia, coronary artery disease, congestive heart failure, peripheral artery disease, ischemic stroke, atrial fibrillation, chronic kidney disease, and telehealth program. The assumption of proportional hazard was tested by the Schoenfeld partial residuals, in which the study group was the only explanatory continuous variable. The assumption of proportional hazard was not rejected. The Bonferroni correction was used to adjust for multiple (pairwise) comparisons in the study group when the overall test was statistically significant. Data were analyzed using Statistical Package for Social Science (version 22; IBM Corp, Armonk, NY). Statistical significance was set at two-sided *P* values <.05.

## Results

### Patient Demographics and Clinical Features

**Figure 1:**
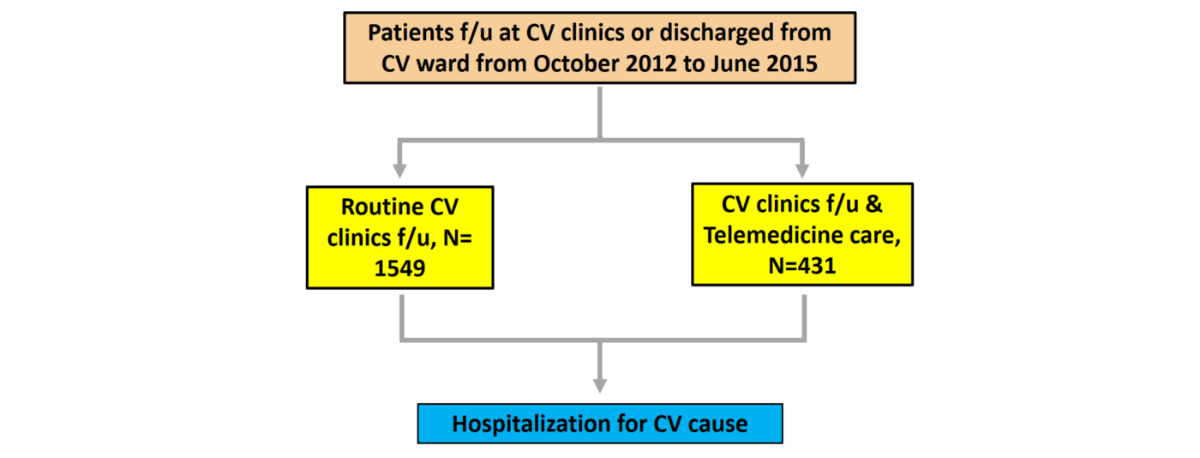
Flow chart of enrolled patients. CV: cardiovascular; f/u: follow up.

A total of 1980 patients (431 in the telehealth group and 1549 in the control group) were enrolled in this study (Figure 1). The baseline characteristics are reported in the Table 1. In the telehealth groups, the mean age was 70.3 (SD 14.9) years, and 66.1% (285/431) were men.

**Table 1:** Baseline characteristics.

Characteristics		Telehealth care group (N=431)	Control group (N=1549)	*P* value
**Patient status**				
	Age (years), mean (SD)	70.3 (14.9)	64.2 (13.8)	<.001
	Age > 80 years, n (%)	85 (19.7)	115 (7.4)	<.001
	Female gender, n (%)	146 (33.9)	494 (31.9)	.45
**Risk factors**				
	Diabetes mellitus, n (%)	128 (29.7)	369 (23.8)	.01
	Hypertension, n (%)	198 (45.9)	642 (41.4)	.098
	Hyperlipidemia, n (%)	143 (33.2)	484 (31.2)	.40
	Atrial fibrillation, n (%)	73 (16.9)	152 (9.8)	<.001
	CKD^a^, n (%)	38 (8.8)	116 (7.5)	.36
	CAD^b^, n (%)	207 (48.0)	907 (58.6)	<.001
	CHF^c^, n (%)	103 (23.9)	166 (10.7)	<.001
	Stroke, n (%)	64 (14.8)	128 (8.3)	<.001
	PAD^d^, n (%)	14 (3.2)	51 (3.3)	>.99
	CHA_2_DS_2_-VASc^e^ score, mean (SD)	2.7 (1.9)	2.0 (1.6)	<.001
**Medication**				
	Aspirin, n (%)	184 (42.7%)	788 (50.9%)	.003
	Beta-blocker, n (%)	150 (34.8%)	321 (20.7%)	<.001
	ACEI/ARB^f^, n (%)	170 (39.4%)	510 (32.3%)	.01
	CCB^g^, n (%)	44 (10.2%)	390 (25.2%)	<.001
	Statin, n (%)	120 (27.8%)	440 (28.4%)	.86
	OHA^h^, n (%)	78 (18.1%)	229 (14.8%)	.098
	Mean follow-up (days), mean (SD)	701.7 (431.4)	886.1 (530.9)	<.001

^a^CKD: chronic kidney disease.

^b^CAD: coronary artery disease.

^c^CHF: congestive heart failure.

^d^PAD: peripheral artery disease.

^e^CHA_2_DS_2_-VASc: congestive heart failure, hypertension, age >75 years (doubled), type 2 diabetes mellitus, previous stroke, transient ischemic attack or thromboembolism (doubled), vascular disease, age of 65-75 years, and sex.

^f^ACEI/ARB: angiotensin-converting enzyme inhibitor/angiotensin II receptor blocker.

^g^CCB: calcium channel blocker.

^h^OHA: oral hypoglycemic agent.

In the control group, the mean age was 64.2 (SD 13.8) years, and 68.11% were men (1055/1549). The telehealth group had more patients with congestive heart failure (103/431, 23.9% vs 166/1549, 10.7%), stroke (64/431, 14.8% vs 128/1549, 8.3%), diabetes mellitus (128/431, 29.7% vs 369/1549, 23.8%), and atrial fibrillation (73/431, 16.9% vs 152/1549, 9.8%) than the control group; all these variables were significantly different between the two groups. The CHA_2_DS_2_-VASc score was significantly higher in the telehealth group than in the control group (2.7 [SD 1.9] vs 2.0 [SD 1.6], *P*<.001). The mean follow-up time was 701.7 (SD 431.4) days for the telehealth group and 886.1 (SD 530.9) days for the control group (*P*<.001).

### Prognosis of Patients Stratified by CHA _2_DS_2_-VASc Score

**Figure 2:**
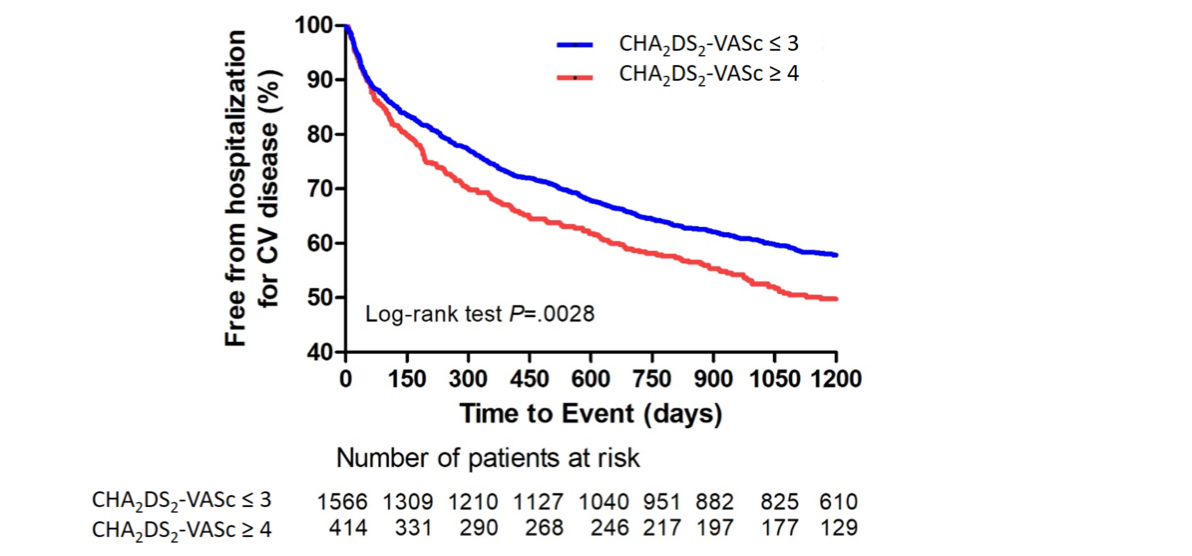
Kaplan-Meier curve of cardiovascular hospitalization according to CHA_2_DS_2_-VASc scores. The overall estimated rate of cardiovascular hospitalization was 54.8% and 46.5% in patients with CHA_2_DS_2_-VASc score ≤3 and ≥4, respectively (log-rank test *P*=.003). The dotted lines represented the error bars of 95% CI in both figures. CV: cardiovascular; CHA_2_DS_2_-VASc: congestive heart failure, hypertension, age >75 years (doubled), type 2 diabetes mellitus, previous stroke, transient ischemic attack or thromboembolism (doubled), vascular disease, age of 65-75 years, and sex.

We stratified all patients (telehealth and control group) into higher (4-8 points) and lower (0-3 points) score groups according to their CHA_2_DS_2_-VASc score. A total of 414 patients were included in the higher score group and 1566 were included in the lower score group. The Kaplan-Meier curve showed significant differences in the factor remaining free of hospitalization for a cardiovascular event between the two groups. The overall estimated survival rate was 46.5% in the higher CHA_2_DS_2_-VASc score group and 54.8% in the lower CHA_2_DS_2_-VASc score group (log-rank test *P*=.003; Figure 2).

### Impact of Telehealth Program on Patients with Different CHA _2_DS _2_-VASc Scores

**Figure 3:**
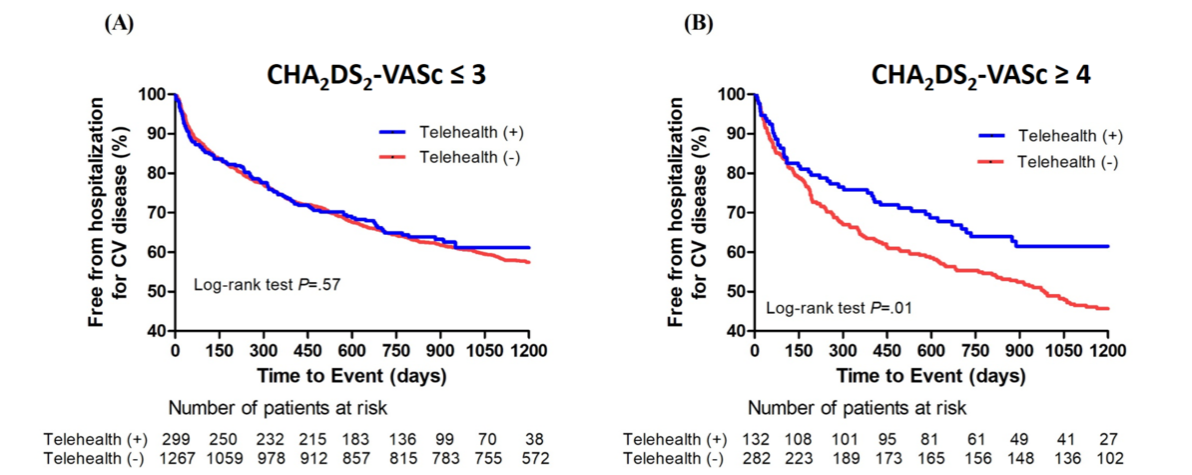
(A) Kaplan-Meier curve of cardiovascular hospitalization and the fourth generation synchronous telehealth program in patients with CHA_2_DS_2_-VASc score ≤3. The overall estimated survival rate of remaining free from cardiovascular hospitalization was 61.1% in patients accepting the fourth-generation telehealth program, and 54.3% in patients not accepting the program (log-rank test *P*=.57). (B) CHA_2_DS_2_-VASc score ≥4. The overall estimated survival rate of remaining free from cardiovascular hospitalization was 61.5% in patients accepting the fourth-generation telehealth program and 41.8% in patients not accepting the program (log-rank test *P*=.01). CV: cardiovascular; CHA_2_DS_2_-VASc: congestive heart failure, hypertension, age >75 years (doubled), type 2 diabetes mellitus, previous stroke, transient ischemic attack or thromboembolism (doubled), vascular disease, age of 65-75 years, and sex.

The lower CHA_2_DS_2_-VASc score group included 299 subjects in the telehealth group and 1267 subjects in the usual care group. The estimated survival rate of patients remaining free from cardiovascular hospitalization was 61.4% for the telehealth program and 54.3% in the control group. The Kaplan-Meier curve showed similar survival rates in both groups without significant differences (log-rank *P*=.57; Figure 3). The higher CHA_2_DS_2_-VASc score group included 132 subjects in the telehealth group and 282 in the usual care group. The estimated survival rate in patients remaining free from cardiovascular hospitalization was 61.5% and 41.8% in patients in the telehealth program and those in the control group, respectively. Patients accepting the telehealth program had better survival, with significant differences observed in the Kaplan-Meier curve between the two groups (log-rank *P*=.01; Figure 3).

### Predictors of Cardiovascular Hospitalization for Patients With CHA_2_DS_2_-VASc Scores ≥4

Univariate analysis revealed a significant association between hospitalization for cardiovascular events and the following variables in patients with CHA_2_DS_2_-VASc scores ≥ 4 (Table 2): chronic kidney disease (hazard ratio [HR]=1.87 [CI 1.32-2.63], *P*<.001), telehealth program (HR=0.66 [CI 0.48-0.91], *P*=.01), and angiotensin receptor blocker (HR=0.70 [CI: 0.52-0.94], *P*=.006). However, only chronic kidney disease (HR=1.65 [CI 1.13-2.40], *P*=.01) and the telehealth program (HR=0.36 [CI 0.22-0.62], *P*<.001) remained significant in multivariable Cox regression. On the other hand, the variable peripheral artery disease was insignificant in univariable analysis (HR=0.97 [CI 0.74-1.29], *P*=.86) but became significant (HR=1.92 [CI 1.24-2.98], *P* *=*.003) after multivariable Cox regression (Table 2).

### Interaction Between the Telehealth Program and the CHA_2_DS_2_-VASc Score

**Figure 4:**
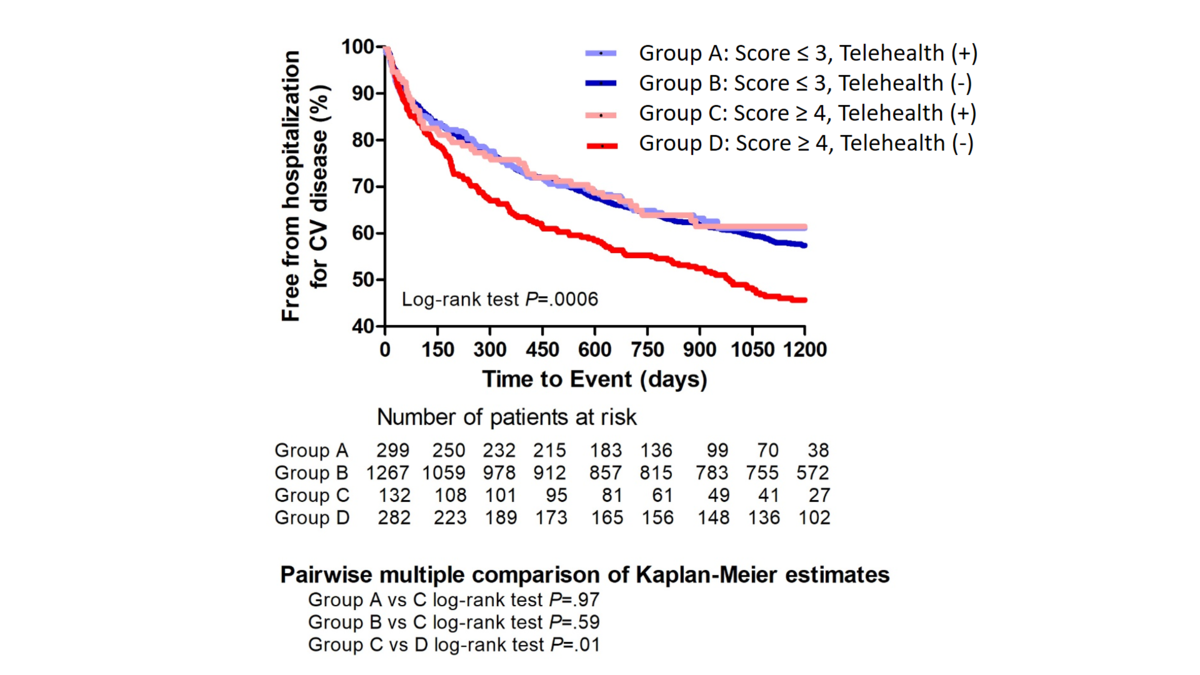
Kaplan-Meier curve of cardiovascular hospitalization in patients with different CHA_2_DS_2_-VASc scores (≥4 and ≤3) with/without the fourth-generation synchronous telehealth program. In the usual care group, the overall estimated survival rate of free from cardiovascular hospitalization was 41.8% in group D and 54.3% in group B. In the telehealth group, the overall estimated survival rate while remaining free of cardiovascular hospitalization was 61.5% in group C and 61.1 % in group A (log-rank test *P*=.0006). After pairwise multiple comparison adjustment procedure for Kaplan-Meier survival curve with Bonferroni correction, the estimated survival rate of group C remained significantly higher than that of group D. It was similar in groups A and B (Group A vs C: log-rank test *P*=.97; Group B vs C: log-rank test *P*=.59; Group C vs D: log-rank test *P*=.01). CV: cardiovascular; CHA_2_DS_2_-VASc: congestive heart failure, hypertension, age >75 years (doubled), type 2 diabetes mellitus, previous stroke, transient ischemic attack or thromboembolism (doubled), vascular disease, age of 65-75 years, and sex.

Figure 4 shows the Kaplan-Meier curve of cardiovascular hospitalization among patients with different CHA_2_DS_2_-VASc scores (≥4 and ≤3) who did and did not receive the fourth-generation synchronous telehealth program. In the usual care group, the overall estimated survival rate of patients who were not hospitalized for cardiovascular complications was 41.8% in group D and 54.3% in group B (Figure 4). In the telehealth group, the overall estimated survival rate of subjects remaining free from cardiovascular hospitalization was 61.5% in group C and 61.1% in group A (log-rank *P*=.0006) After a pairwise multiple comparison-adjustment procedure for Kaplan-Meier survival curve with Bonferroni correction, the estimated survival rate of group C remained significantly higher than that for group D and was similar to that of groups A and B (group A vs group C, log-rank *P*=.97; group B vs group C, log-rank *P*=.59; group C vs group D, log-rank *P*=.01; Figure 4).

**Table 2:** Univariate and multivariable Cox analyses: Predictors of hospitalization for cardiovascular events in patients with CHA_2_DS_2_-VASc scores ≥4 (N=414).

Variables	Univariate analysis		Multivariable analysis	
	Hazard ratio (95% CI)	*P* value	Hazard ratio (95% CI)	*P* value
Age >80 years	0.85 (0.65-1.12)	.26	0.93 (0.69-1.23)	.59
Female gender	0.86 (0.66-1.14)	.30	0.97 (0.73-1.30)	.85
Diabetes mellitus	0.99 (0.75-1.30)	.93	0.85 (0.60-1.22)	.39
Hypertension	1.05 (0.73-1.50)	.81	1.35 (0.91-2.02)	.14
Dyslipidemia	1.07 (0.82-1.41)	.62	0.94 (0.69-1.30)	.72
Coronary artery disease	1.30 (0.98-1.73)	.07	1.22 (0.88-1.68)	.23
Congestive heart failure	1.24 (0.93-1.66)	.14	1.31 (0.96-1.80)	.09
Peripheral artery disease	0.97 (0.74-1.29)	.86	1.92 (1.24-2.98)	.003
Ischemic stroke	0.76 (0.40-1.32)	.39	0.84 (0.44-1.61)	.60
Atrial fibrillation	1.03 (0.73-1.46)	.86	1.13 (0.78-1.64)	.51
Chronic kidney disease	1.87 (1.32-2.63)	<.001	1.65 (1.13-2.40)	.01
Telehealth program	0.66 (0.48-0.91)	.01	0.36 (0.22-0.62)	<.001
Antiplatelet	1.03 (0.78-1.35)	.86	1.03 (0.75-1.41)	.86
Angiotensin converting enzyme inhibitor	0.99 (0.54-1.83)	.99	0.96 (0.50-1.82)	.89
Angiotensin II receptor blocker	0.70 (0.52-0.94)	.02	0.80 (0.56-1.17)	.25
Calcium channel blocker	0.76 (0.55-1.05)	.098	0.75 (0.53-1.06)	.10
Beta-blocker	1.02 (0.74-1.40)	.92	1.06 (0.75-1.49)	.76
Statin	0.93 (0.69-1.27)	.65	1.08 (0.74-1.57)	.69
Oral hypoglycemic agent	0.75 (0.55-1.04)	.08	1.00 (0.65-1.53)	.99
Insulin	0.80 (0.44-1.47)	.47	0.97 (0.51-1.84)	.92

## Discussion

### Principal Findings

This is the first study to apply CHA_2_DS_2_-VASc scores to stratify patients receiving the fourth-generation synchronous telehealth program and determine who benefits most from the program. Our study showed that the fourth-generation synchronous telehealth program provided better outcomes with reduced cardiovascular hospitalization than usual care in patients with higher CHA_2_DS_2_-VASc scores (≥4 points).

### Overview

Telehealth care has been shown to reduce hospitalizations in patients with chronic conditions such as asthma, chronic obstructive pulmonary disease, and heart failure [13-16]. We previously reported better cost effectiveness and clinical outcomes with the use of a fourth-generation synchronous telehealth program in patients with chronic cardiovascular diseases [12]. In that study, patients who received and those who did not receive telehealth programs were matched for sex, age, and Charlson score, which is a method of predicting mortality by classifying or weighting comorbid conditions [17]. However, some studies failed to show better clinical outcomes in patients receiving telehealth program [6,7]. Although the types of subjects enrolled were diverse, research on telehealth programs in chronic diseases management has shown mixed results. Development of an objective stratification system for patients and identification of which group of patients benefit from the telehealth program are needed.

The CHA_2_DS_2_-VASc score is a validated clinical tool predicting stroke occurrence in patients with atrial fibrillation [8,9]. Previous studies have shown that the CHA_2_DS_2_-VASc score enables a substantially comprehensive risk evaluation and improves physicians’ ability to identify genuinely low-risk patients who have atrial fibrillation. It is not surprising that the components of these risk scores are associated with adverse outcomes because the majority of variables reflect the presence of heart disease or heart disease risk factors independent of atrial fibrillation. These grave outcomes are highlighted in studies that have examined CHA_2_DS_2_-VASc scores in patients without atrial fibrillation. These studies have noted a significant risk of major adverse cardiovascular events with increasing scores [18-20]. Comprehensive care for a patient with higher scores is extremely important because they have a greater risk and worse outcomes of congestive heart failure, acute coronary syndrome, and even mortality [18-20]. In this study, we did not match both groups by the Charlson score: Several components of CHA_2_DS_2_-VASc and Charlson scores were repetitive. Since patients with higher CHA_2_DS_2_-VASc scores exhibited a higher prevalence of cardiovascular diseases, the CHA_2_DS_2_-VASc score may be a better parameter than the Charlson score to select potential candidates for telehealth care.

In patients with nonvalvular atrial fibrillation, the CHADS_2_ score was significantly associated with the risk of a future stroke event. This new scoring system can identify patients with lower CHADS_2_ scores (≤1) who remain at high risk for stroke. However, Eva et al reported that the demarcation may be minimal for patients with CHA_2_DS_2_-VASc scores ≤3 [21]. We apply this result to our patients in two groups, with higher (≥4) and lower (≤3) CHA_2_DS_2_-VASc scores. In this study, the two groups had a significant difference in survival of subjects remaining free from cardiovascular hospitalization overall. More importantly, we found that this demarcation can stratify whether patients receive benefits after accepting the telehealth program.

In the usual care group, we found that a higher CHA_2_DS_2_-VASc score (≥4) is significantly associated with a higher risk of cardiovascular admission compared to a lower score (≤3) (Multimedia Appendix 1). However, the cardiovascular admissions were similar for patients with CHA_2_DS_2_-VASc scores ≥4 and those with scores ≤3 in the telehealth group (Multimedia Appendix 1). This suggested that telehealth care could diminish the CHA_2_DS_2_-VASc score-associated cardiovascular admission. In the patient group with CHA_2_DS_2_-VASc scores ≤3, the overall estimated survival rate of subjects remaining free from cardiovascular hospitalization was similar irrespective of whether patients accepted the fourth-generation telehealth program (Figure 3). However, patients with scores ≥4 who accepted the telehealth program had significantly better outcome than those who did not accept the program (Figure 3B). After multiple comparison adjustments by Bonferroni methods, patients with scores ≥4 accepting telehealth were found to have similar clinical outcomes as patients with scores ≤3, regardless of whether they accepted the telehealth program; their outcomes were significantly better than those of patients with scores ≥4 not accepting the telehealth program (Figure 4). This implied that patients with scores ≥4 benefit most from telehealth monitoring and the need for cardiovascular hospitalization is reduced. These results showed that the CHA_2_DS_2_-VASc score might be a good indicator to select patients for the telehealth program.

The telehealth program has changed over time. We used the fourth-generation telehealth program, which is a synchronous and integrated remote-management program for chronic disease. This new system takes the initiative to offer an interactive environment and in-time responsiveness for patients who encountered acute illness or deterioration in condition. Compared to usual care, more accurate diagnoses and decisions can be made through quick communication after accepting the telehealth program. In our previous report, there were significantly fewer emergency department visits, hospitalizations, hospitalization days, and intensive care unit admissions per month in the telehealth group compared to the control group [12]. Thus, this new intervention program may be helpful in improving patient outcomes.

Apart from the telehealth program, we found that chronic kidney disease and peripheral artery disease were the two remaining prognostic factors for cardiovascular admission in patients with CHAS_2_DS_2_-VASc scores ≥ 4 after multivariable Cox regression. Studies on patients with chronic kidney disease accepting telehealth are rare [22,23]. We reported that among patients receiving the telehealth program, renal function status remains a predictor for first hospitalization—this is identical to our study [24]. On the other hand, patients with peripheral artery disease accepting the telehealth program are rarer despite their higher mortality rate [25]. According to previous studies, the mortality of symptomatic and asymptomatic patients was 19% and 24%, respectively, at 5 years [26]. Furthermore, patients with peripheral artery disease share similar risk factors as patients with coronary artery disease, carotid artery stenosis, and congestive heart failure as compared to the general population [27-29]. Early identification of peripheral artery disease with optimized and comprehensive treatment is mandatory to improve clinical outcomes; the telehealth program may have a role in this process. There have been studies addressing the issue of telehealth applications in patients with peripheral artery disease with wounds or gangrene, but these studies focused on wound infection control rather than general patient care [30,31]. Larger clinical trials applying telehealth care to patients with peripheral artery disease may be needed to improve clinical outcomes.

### Limitations

This study has several limitations. First, the study was not randomized, which resulted in heterogeneity of the patient population, disease severity, and patient selection. Patients with the capacity to self-monitor and be enrolled in a program for home remote monitoring are potentially more likely to receive medical assistance when experiencing clinical changes or a clinical decline. The patient-selection process should be recognized as a limitation. Second, the presence of numerous confounding factors in our cohort might have influenced the result, including the missing events. We tried to perform multivariable Cox regression analysis to minimize the possible confounding effect of other clinical factors. Third, there might be some statistical limitations and considerations of statistical testing/modeling. For example, peripheral artery disease was not significantly associated with hospitalization for cardiovascular events in the univariate Cox regression but became significant in the multivariable Cox model in patients with CHA_2_DS_2_-VASc scores ≥4 (Table 2). This might be due to some reverse confounding or overfitting. On the other hand, the survival analysis stratified by CHA_2_DS_2_-VASc scores (≤3 vs >4) may induce type I error inflation (Figure 3). However, the result would be significant even if Bonferroni correction was done in patients with CHA_2_DS_2_-VASc scores ≥4. This indicates that type I error inflation may not be a serious problem in this study. Fourth, the clinical outcomes were derived from the electronic billing and medical records of our hospital, and the patients who received care outside our hospital were not recorded. Resources that were used but not billed may have also been overlooked when extracting data from our billing system.

### Conclusions

Patients with higher CHA_2_DS_2_-VASc scores had higher risks of cardiovascular admissions, but the fourth-generation telehealth program could diminish the outcome difference associated with scores. Patients with CHA_2_DS_2_-VASc scores ≥4 benefited the most from the fourth-generation telehealth program and remained free of cardiovascular hospitalization.

## Appendix

Multimedia Appendix 1 (A) Kaplan-Mayer curve of cardiovascular hospitalization with CHA_2_DS_2_-VASc score in patients who did not accept the fourth-generation synchronous telehealth program. The overall estimated survival rate of cardiovascular hospitalization was 41.8% in patients with CHA_2_DS_2_-VASc score ≥4 and 54.3% in patients with a score ≤3 (log-rank test *P*=.0001). (B) Patients using the fourth-generation synchronous telehealth program. The overall estimated survival rate of cardiovascular hospitalization was 61.5% in patients with CHA_2_DS_2_-VASc score ≥4 and 61.1% in patients with the score ≤3 (log-rank test *P*=.97). CV: cardiovascular; CHA_2_DS_2_-VASc: congestive heart failure, hypertension, age > 75 years (doubled), type 2 diabetes mellitus, previous stroke, transient ischemic attack or thromboembolism (doubled), vascular disease, age of 65-75 years, and sex.

## Abbreviations

ACEI: angiotensin converting enzyme inhibitor
ARB: angiotensin II receptor blocker
CAD: coronary artery disease
CCB: calcium channel blocker
CHA_2_DS_2_-VASc: congestive heart failure, hypertension, age >75 years (doubled), type 2 diabetes mellitus, previous stroke, transient ischemic attack or thromboembolism (doubled), vascular disease, age of 65-75 years, and sex
CHF: congestive heart failure
CKD: chronic kidney disease
HR: hazard ratio
OHA: oral hypoglycemic agent
PAD: peripheral artery disease
