# Supplementary figures and images for: Use of the CHA2DS2-VASc Score for Risk Stratification of Hospital Admissions Among Patients With Cardiovascular Diseases Receiving a Fourth-Generation Synchronous Telehealth Program: Retrospective Cohort Study
Source: J Med Internet Res. 2019 Jan 31;21(1):e12790. doi: 10.2196/12790 (PMC6374726; doi:10.2196/12790)

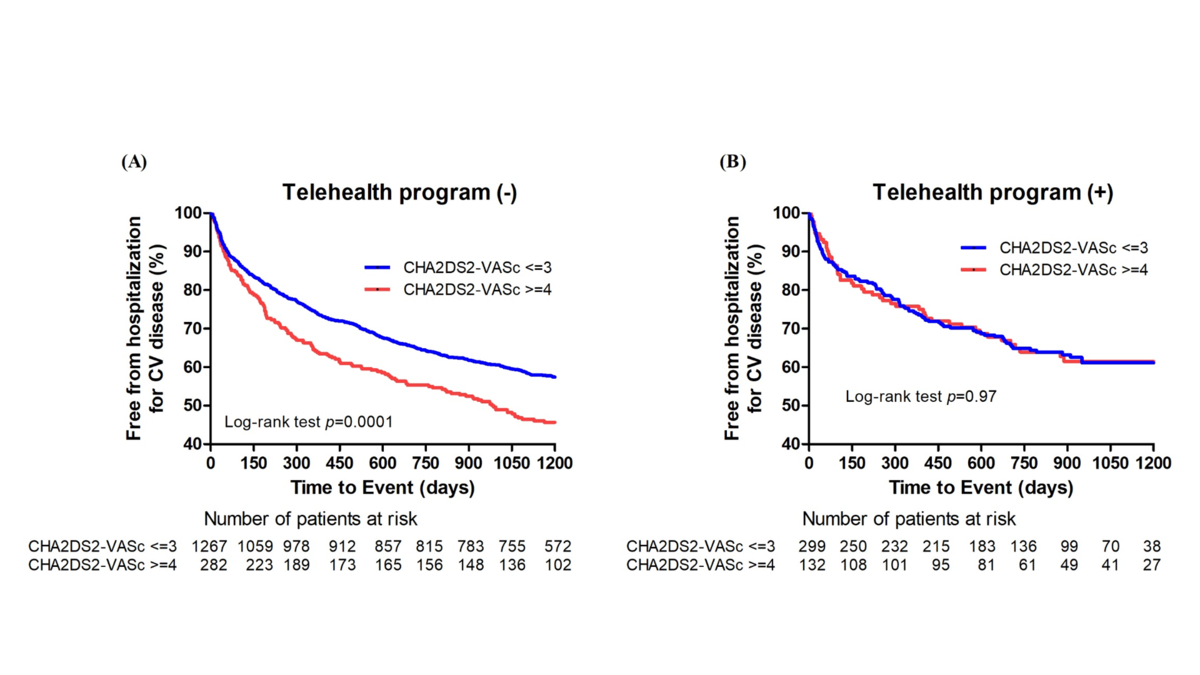

Supplement: Multimedia Appendix 1 [file jmir_v21i1e12790_app1.png]
